# Engineering of Substrate-Binding Domain to Improve Catalytic Activity of Chondroitin B Lyase with Semi-Rational Design

**DOI:** 10.3390/cimb46090591

**Published:** 2024-09-06

**Authors:** Miao Tian, Yuan-Yuan Xu, Yang-Nan Li, Shen Yu, Yi-Lin Wang, Xiao-Lai Ma, Ye-Wang Zhang

**Affiliations:** 1 School of Pharmacy, Jiangsu University, Zhenjiang 212013, China; m_tian_ujs@163.com (M.T.); xuyuanyuanmail@yeah.net (Y.-Y.X.); liyangnanlight@outlook.com (Y.-N.L.); yushen.edu@outlook.com (S.Y.); wyl0004@sohu.com (Y.-L.W.); 2 School of Pharmacy, Guilin Medical University, Guilin 541004, China

**Keywords:** dietary supplement, chondroitin B lyase, catalytic activity, structural modification, molecular docking

## Abstract

Dermatan sulfate and chondroitin sulfate are dietary supplements that can be utilized as prophylactics against thrombus formation. Low-molecular-weight dermatan sulfate (LMWDS) is particularly advantageous due to its high absorbability. The enzymatic synthesis of low-molecular-weight dermatan sulfates (LMWDSs) using chondroitin B lyase is a sustainable and environmentally friendly approach to manufacturing. However, the industrial application of chondroitin B lyases is severely hampered by their low catalytic activity. To improve the activity, a semi-rational design strategy of engineering the substrate-binding domain of chondroitin B lyase was performed based on the structure. The binding domain was subjected to screening of critical residues for modification using multiple sequence alignments and molecular docking. A total of thirteen single-point mutants were constructed and analyzed to assess their catalytic characteristics. Out of these, S90T, N103C, H134Y, and R159K exhibited noteworthy enhancements in activity. This study also examined combinatorial mutagenesis and found that the mutant H134Y/R159K exhibited a substantially enhanced catalytic activity of 1266.74 U/mg, which was 3.21-fold that of the wild-type one. Molecular docking revealed that the enhanced activity of the mutant could be attributed to the formation of new hydrogen bonds and hydrophobic interactions with the substrate as well as neighbor residues. The highly active mutant would benefit the utilization of chondroitin B lyase in pharmaceuticals and functional foods.

## 1. Introduction

Chondroitin lyases are enzymes that can cleave the O-glycosidic bonds of glycosaminoglycans (GAGs), such as chondroitin sulfate/dermatan sulfate (CS/DS) and hyaluronic acid (HA) [1,2,3]. The enzymes are classified into chondroitin ABC, AC, and B lyases based on the composition of their substrates [4,5,6,7,8]. Chondroitin B lyase has the ability to selectively cleave β-1,4 glycosidic linkages between N-Acetyl-D-galactosamine (GalNAc) and L-Iduronic acid (L-IdoA), resulting in the production of low-molecular-weight dermatan sulfate (LMWDS), which is easily absorbed and exhibits enhanced biological efficacy in stimulating bone formation, influencing neural maturation, and modulating inflammatory responses to impede the growth of nerve fibers [9,10,11,12,13,14]. LMWDS can be used as both an anticoagulant and a nutritional supplement for managing and preventing conditions like arthritis [15,16]. It has been demonstrated that the oligosaccharide-structured CS/DS improves the absorption of iron in skimmed milk and has promising uses in dairy nutrition [17]. Furthermore, chondroitin B lyases can be applied to detect or remove DS impurities from heparin, leading to its potential use in the production of heparin [18]. Enzymatic reactions with chondroitin B lyase are mild and significantly more effective than the traditional physical or chemical degradation of DS, making it the most promising approach for the preparation of LMWDS. The priority of the enzymatic process is the highly active and stable enzyme used in industry or clinics [19,20,21]. However, the enzyme activity of chondroitin B lyases derived from *Flavobacterium heparinum* was only 84 U/mg [4,22]. Similarly, the specific activity of EnCSase from *Photobacterium* sp. for DS was 141 U/mg [23]. These enzymes cannot fit the industrial requirements, suffering from low catalytic activity. Thus, it is imperative and time-sensitive to conduct research on the screening of novel enzymes or the modification of the reported chondroitin B lyase that has potential industrial applications.

A protein engineering strategy has been applied to improve enzyme performance, including the glycosaminoglycan lyases [24,25,26,27]. It is reported that heparinase I could be engineered to significantly improve its activity and thermal stability. A single-point mutant Q157H showed 6.0-fold half-lives and 1.88-fold enzyme activity of the wild-type one [28]. The same group then engineered the substrate and Ca^2+^ binding domains of heparinase I, and the mutant D152S/R244K/T250D was constructed, expressed, and characterized to have a 5.26-fold catalytic efficiency of the wild-type one [29]. The application of protein engineering strategy to chondroitin AC lyase also significantly enhances its properties. By engineering the terminal region of chondroitin AC lyase, mutants K43G and S673V showed 95.0% and 193.8% half-lives of the wild type at 37 °C, respectively, and 39.7% and 27.13% increase in catalytic activity [30]. Semirational design is a practical strategy in protein engineering that utilizes computer-assisted targeted mutagenesis of crucial amino acid residues near the active site based on the researchers’ experience [31,32,33]. The catalytic activity of HpfutC was improved 2.3 times by applying saturation mutagenesis and combinatorial mutagenesis to nonconserved regions as a part of a semirational design [34].

Previously, a highly active chondroitin B lyase from *Pedobacter schmidteae* (*Ps*ChonB) was cloned and expressed as a soluble protein heterologous in *Escherichia coli* (*E. coli*) [35]. To enhance its catalytic efficiency, a semirational design combination of sequence alignment and structural analysis of the substrate-binding domain was employed in the present work. Molecular docking was further performed to elucidate the interactions between the enzyme and substrate and reveal the possible reasons for the increased catalytic activity.

## 2. Materials and Methods

### 2.1. Materials

SanPrep Mini Plasmid DNA extraction kit, *E. coli* DH5α, and BL21 (DE3) competent cells were purchased from Sangon Biotech (Shanghai, China). NaOH, Tris, anhydrous ethanol, tryptone, acrylamide, ammonium persulfate (APS), ethylenediaminetetraacetic acid (EDTA), imidazole, bromophenol blue, glycine, sodium dodecyl sulfate (SDS), mercaptothion, kanamycin, yeast extract, NaCl, HCl, and CaCl_2_ were bought from Sinopharm (Shanghai, China). Isopropyl-β-thiogalactopyranoside (IPTG) and bovine serum protein (BSA) were purchased from Aladdin (Shanghai, China). Dermatan sulfate was supplied by Sigma (Shanghai, China).

### 2.2. Homologous Modeling and Molecular Docking

Based on the high similarity (83.8%) and homology (72.3%) between *Ps*ChonB and chondroitin B lyase (*Ph*ChonB, PDB code: 1ofl) from *Pedobacter heparinus*, the theoretical structure of *Ps*ChonB was constructed with *Ps*ChonB as the template, using the online tool SWISS-MODEL (https://swissmodel.expasy.org, accessed on 12 January 2022). The quality of the constructed *Ps*ChonB model was verified using the SAVES (https://saves.mbi.ucla.edu/, accessed on 31 January 2022). The structure of the ligand was extracted from the complex structure of *Ph*ChonB (PDB code: 1ofl). For *Ps*ChonB and mutants, the ligands were semiflexible docked via AutoDock 4.2.6 with the gate position set to 0.375 Å and the gate number and algorithm parameters set to default values. The coordinates of the box center were set as x = 26, y = 23, and z = 0.69; and the box size was set as 50 × 50 × 50 Å. The binding between substrate and enzyme and the interactions were displayed with PyMOL (http://www.pymol.org, accessed on 18 May 2022). The dermatan sulfate planar structure is from NCBI-PubChem (https://pubchem.ncbi.nlm.nih.gov/, accessed on 12 May 2022).

### 2.3. Construction of the Mutation Library

The amino acid sequence of *Ps*ChonB was analyzed using BLASTp (https://blast.ncbi.nlm.nih.gov/, accessed on 5 July 2022). The multiple sequence alignments of chondroitin B lyases from various organisms were conducted using ClustalW (https://www.genome.jp/tools-bin/clustalw, accessed on 10 August 2022) and visualized with espript3.0 (https://espript.ibcp.fr/ESPript/, accessed on 10 August 2022). The mutation library was constructed by combining the results of molecular docking and consensus design.

### 2.4. Mutagenesis of PsChonB

The mutagenesis of *Ps*ChonB was performed with the mutation kits by following the protocol. The volume of the amplification reaction system was 50 µL, which contained *Ps*ChonB plasmid with a total molecular weight of 1 ng (1 µL), dNTPs (1 µL), and forward and reverse primers with a total molecular weight of 10 mM (1 µL), and the sequences of the mutant primers are shown in Table S1, PCR buffer containing Mg^2+^ (5 µL) and Pfu DNA polymerase (1.2 µL), and the remaining components were ddH_2_O water. The PCR cycling program was set by reference to the parameters in the targeted mutation kit, and the annealing temperatures were changed according to *T_m_* values of the reference primers. The annealing temperatures are shown in Table S1, and the extension time was set to 14.4 min. The PCR products were digested with DpnI for 1 h at 37 °C and then transformed into *Escherichia coli* (*E. coli*) competent cells after the confirmation of the sequence.

### 2.5. Expression and Purification of PsChonB and Mutants

Recombinant *E. coli* strains harboring the recombinant plasmid of *Ps*ChonB or the mutants were cultured in 5 mL LB medium containing kanamycin (50 mg mL^−1^) for 10 h at 37 °C. They were then transferred to 100 mL LB medium and incubated at 37 °C until the optical density at 600 nm reached 0.8, and 0.01 mM isopropyl-β-D-thiogalactopyranoside (IPTG) was added to induce the expression of the enzymes for 20 h at 15 °C. The harvested cells were collected by centrifugation (5000 rpm, 10 min at 4 °C) and washed three times with 50 mM Tris-HCl containing 20 mM CaCl_2_ and 50 mM NaCl (pH 8.0). Then, the cells were resuspended with the lysis buffer and subjected to ultrasonication for 10 min, while the supernatant containing enzyme protein was obtained by centrifugation at 8000 rpm at 4 °C for 10 min to remove cellular debris. All the enzymes or mutants in the supernatant were bound with 1 mL of Ni-NTA resin for 3 h at 4 °C. After washing with wash buffer (50 mM Tris-HCl, 20 mM CaCl_2_, 50 mM NaCl, pH 8), the recombinant enzymes were eluted from the Ni-NTA column using 250 mM imidazole buffer (50 mM Tris-HCl, 20 mM CaCl_2_, 50 mM NaCl, pH 8). In the whole process, the protein concentration in each sample was determined with the Bradford method [36]. The purified enzyme was analyzed by 12% sodium dodecyl sulfate–polyacrylamide gel electrophoresis (SDS-PAGE).

### 2.6. Assay of Enzyme Activity

To determine the specific activity of *Ps*ChonB, dermatan sulfate was used as the substrate. In the enzymatic reaction, the unsaturated bonds in the produced oligosaccharides have a maximum absorption peak at 232 nm; thus, the activity of the enzyme was calculated by measuring the optical changes at 232 nm. The reaction components for the hydrolysis of dermatan sulfate consisted of 895 μL of buffer solution (100 mM Tris-HCl, pH 8), 5 μL of enzyme solution, and 100 μL of DS (10 mg/mL) in a total volume of 1 mL. The enzyme activity of *Ps*ChonB was defined as the amount of enzyme required to produce 1 μmoL of unsaturated oligosaccharide product per minute at 40 °C.

### 2.7. Kinetic Parameters of PsChonB

Kinetic parameters of *Ps*ChonB were determined by measuring the enzyme activity in 50 mM Tris-HCl buffer (pH 8, 50 mM Na^+^, 20 mM Ca^2+^) at 40 °C with different substrate concentrations ranging from 0 to 2 mM. The *K*_m_ and *V_max_* of all the enzymes were obtained by nonlinear regression of the Michaelis–Menten equation. All the experiments were repeated three times, and the corresponding standard errors were calculated.

## 3. Results

### 3.1. Site-Directed Mutagenesis of PsChonB

**Figure 1:**
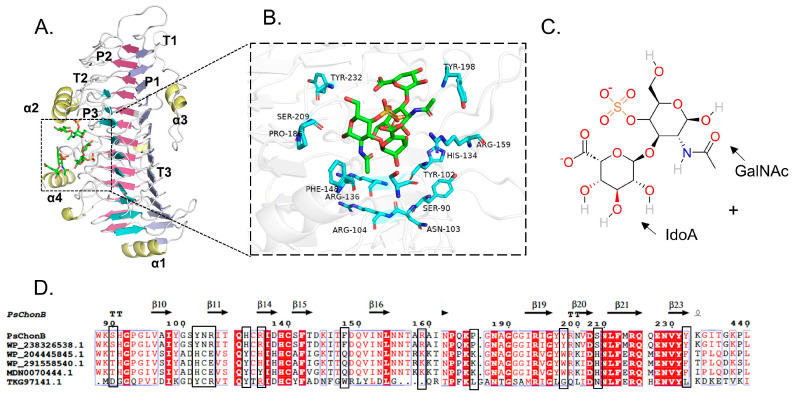
Selection of mutation residues to enhance catalytic activity: (**A**) Structure of *Ps*ChonB. (**B**) Substrate-binding pocket in *Ps*ChonB and the potential mutation residues. (**C**) The 2D structure of dermatan sulfate. (**D**) Multiple sequence alignment of chondroitin sulfate B lyases.

The spatial structure of *Ps*ChonB obtained from the Swiss Model is shown in Figure 1A, and it has a parallel β-helical structure and a right-handed conformation. The cross-section of its β-helical structure shows an L-shape consisting of three β-sheets with different morphologies (P1, P2, and P3) with turns (T1, T2, T3) connecting the β-sheets and four α-helices (α1-α4) covering the surface. The substrate binds within an inverted L-shaped cleft near the bend, which takes the form of coils 7 and 8 of β-sheet P1, α-helix α2, and coils 6–11 extending from the T1 and T3 turns.

The candidate residues located within 5 Å from the substrate-binding domain were screened for mutagenesis if they were not highly conserved amino acid residues (Figure 1B). Subsequently, 13 nonconserved residues, including S90, Y102, N103, R104, H134, R136, F148, R159, P186, Y198, S209, Y232, and G437, were chosen as mutation sites. Based on the result of multiple sequence alignment, the highly conserved amino acid residues were selected for substitution. A small mutant library including S90T, Y102H, N103E, R104E, H134Y, R136H, F148Q, R159K, P186K, Y198W, S209H, Y232F, and G437D was constructed to improve the catalytic activity of *Ps*ChonB (Figure 1D).

### 3.2. Expression of Wild-Type PsChonB and Its Mutants

**Figure 2:**
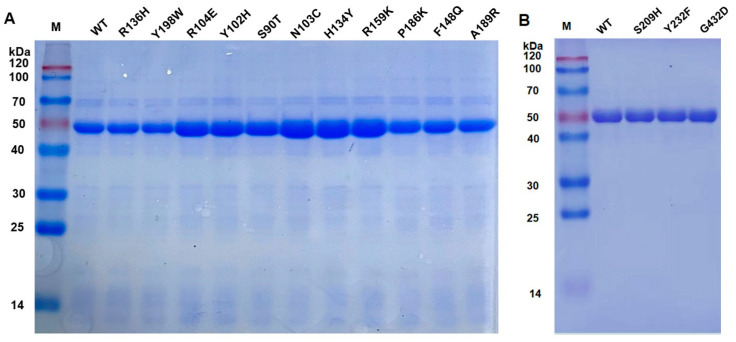
SDS-PAGE images of the wild type and mutants of the single-site mutation. Lane M: protein Marker: (**A**) The lanes from left to right are WT, R136H, R104E, Y102H, S90T, N103C, H134Y, R159K, P186K, F148Q, and A189R. (**B**) The lanes from left to right are WT, S209H, Y232F, and G432D.

All 13 mutants were successfully constructed and expressed heterologously in *E. coli*. The cells containing plasmids of WT (wild type) and mutants were cultured and induced for expression, and all the expressed enzymes were purified with Ni-NTA affinity chromatography and analyzed with SDS-PAGE. As shown in Figure 2, all enzymes, including WT and mutants, showed clear bands at the expected molecular mass of 55 kDa.

### 3.3. Characterization of the PsChonB

**Figure 3:**
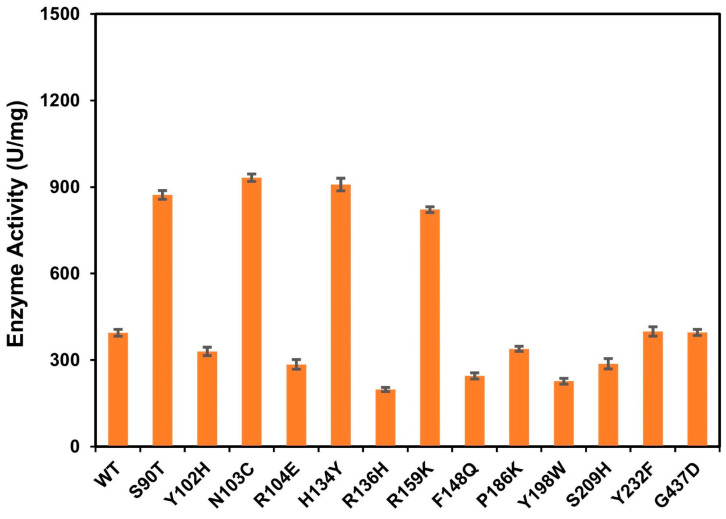
The catalytic activity of *Ps*ChonB and mutants in the ligand-binding domain.

The catalytic activities of WT and its mutants were determined using dermatan sulfate as the substrate. As shown in Figure 3, mutants Y102F, R104E, R136H, F148Q, P186K, Y198W, and S209H showed significant decreases in activity compared with the wild type, with the corresponding decreases of 16.46%, 27.85%, 49.78%, 37.97%, 14.18%, 42.53%, and 27.34%, respectively. The specific activity of mutants Y232F and G437D did not exhibit any noticeable alteration, determining to be 399 U/mg and 396 U/mg, while those of mutants S90T, N103C, H134Y, and R159K displayed significantly improved activities, with 872.9 U/mg, 932.2 U/mg, 908.5 U/mg, and 821.6 U/mg, respectively. The values were 2.21 times, 2.36 times, 2.30 times, and 2.08 times greater than the wild type.

### 3.4. Iterative Mutation and Characterization of the Combinatorial Mutants

**Figure 4:**
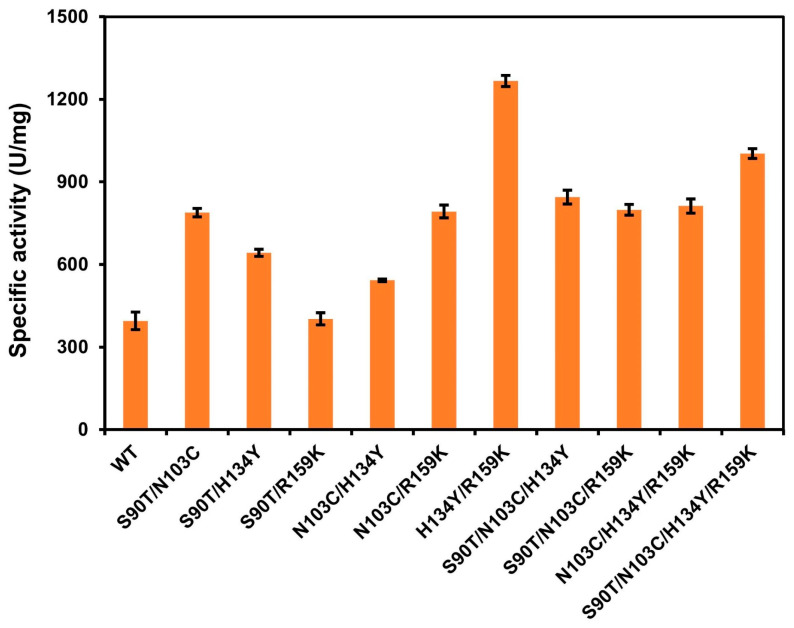
The catalytic activity of combinatorial mutants.

Combined mutations were conducted based on four single mutants with considerably improved activity, and all combination mutants were successfully expressed (Figure S1). The iterative mutants S90T/N103C, S90T/H134Y, S90T/R159K, N103C/H134Y, N103C/R159K, H134Y/R159K, S90T/N103C/H134Y, S90T/N103C/R159K, N103C/H134Y/R159K, and S90T/N103C/H134Y/R159K were measured to be 788.33 U/mg, 646.12 U/mg, 402.31 U/mg, 542.36 U/mg, 792.35 U/mg, 1266.74 U/mg, 844.37 U/mg, 798.54 U/mg, 812.47 U/mg, and 1002.78 U/mg, and increased to 199%, 163%, 102%, 137%, 201%, 321%, 214%, 202%, 206%, and 254% compared with wild type (Figure 4), respectively.

The kinetic characteristics of the single-site mutants that exhibited enhanced activity in the ligand structural domains were determined and compared. Additionally, the combined mutants were also analyzed. The corresponding results are presented in Table 1 and Figure S2. The catalytic efficiencies of single-point mutants S90T, N103C, H134Y, and R159K were significantly increased compared with the wild type, and their *V_max_* were 2.20-fold, 2.35-fold, 2.29-fold, and 2.07-fold higher than that of the wild type, respectively. The iterative mutants S90T/N103C, S90T/H134Y, S90T/R159K, N103C/H134Y, N103C/R159K, H134Y/R159K, S90T/N103C/H134Y, S90T/N103C/R159K, N103C/H134Y/R159K, and S90T/N103C/H134Y/R159K also showed significantly higher catalytic efficiency than the wild type. Additionally, the *V_max_* values of mutants H134Y/R159K and S90T/N103C/H134Y/R159K were 3.42-fold and 2.58-fold higher than those of the wild type, respectively.

### 3.5. Molecular Docking of Enzyme and Substrate

**Figure 5:**
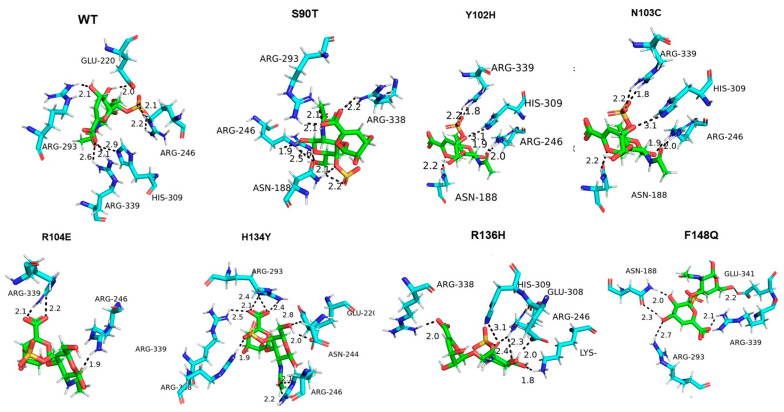

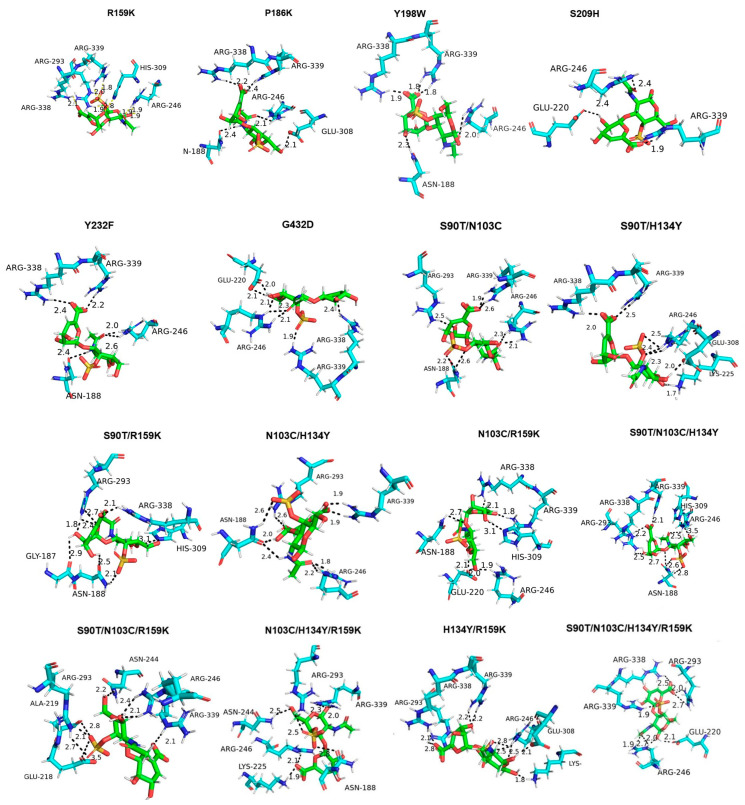
Molecular docking of DS and *Ps*ChonB and the mutants. The substrate is displayed as a red–green complex, with oxygen atoms in red and nitrogen atoms in blue.

In order to explore the reasons for the activity viability of the mutants, molecular docking of the *Ps*ChonB with the dermatan sulfate has been performed (Figure 5). The results indicated that the wild type formed a total of seven hydrogen bonds with the substrate. However, the hydrogen bonds between Y102H, F148Q, R136H, R104E, P186K, Y198W, and S209H and the substrate were noticeably decreased compared with the wild type, with values of 6, 5, 6, 3, 5, 5, and 4, respectively. The mutants S90T, N103C, H134Y, and R159K exhibited 8, 8, 8, and 7 hydrogen bonds, respectively. The average bond lengths for these mutants were 2.25 Å, 2.20 Å, 2.28 Å, and 2.19 Å, which were shorter than the average bond length of the wild type (WT), with 2.45 Å. Upon further analysis of the mutant’s interaction with surrounding amino acids, it was found that S90T exhibited enhanced weak hydrogen bonding with Asp149 and weak interaction bonding with Asn117, as depicted in Figure S3. Additionally, H134Y displayed increased hydrophobic interactions with Tyr102, while R159K exhibited increased hydrophobic interactions with His91 and Tyr197. The mutants S90T, N103C, H134Y, and R159K showed stronger interactions with the substrate with the mutated and the neighboring residues compared with the wild type.

The combinatorial mutants H134Y/R159K, N103C/H134Y, S90T/N103C/H134Y, and S90T/N103C/H134Y/R159K exhibited 9, 8, 8, and 8 hydrogen bonds between enzymes and substrate, respectively. H134Y/R159K, the mutant with the highest specific activity, has an average bond length of 2.33 Å, which is shorter than that of the wild type (WT). The combinatorial mutants S90T/N103C, S90T/H134Y, N103C/R159K, and N103C/H134Y/R159K exhibit the same amount of hydrogen bonds as the wild type (WT). However, their average bond lengths are 2.31 Å, 2.20 Å, 2.24 Å, and 2.30 Å, respectively.

**Figure 6:**
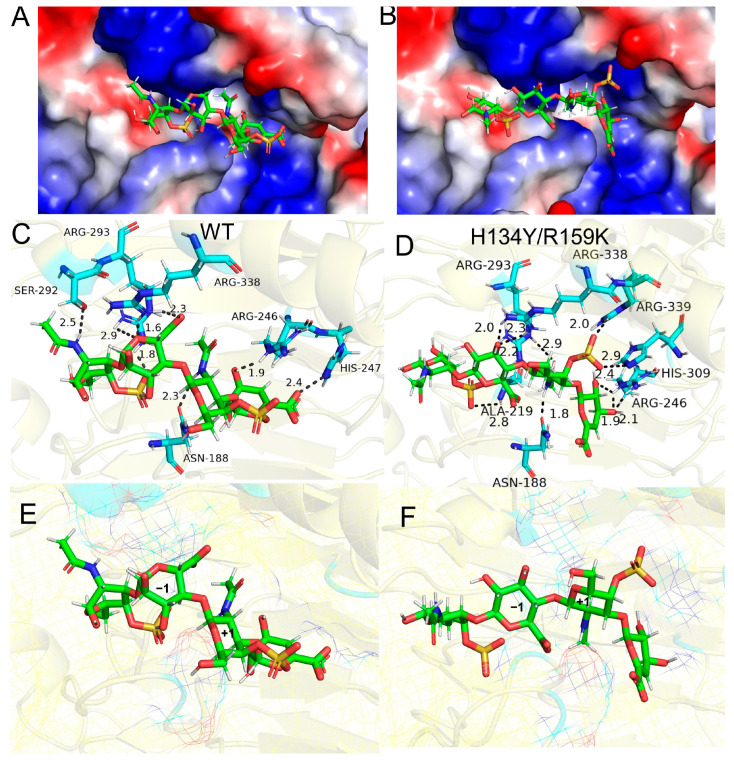
Tetrasaccharide molecular docking of wild type and H134Y/R159K: Surface structures of WT (**A**) and H134Y/R159K (**B**). Molecular docking of WT (**C**) and H134Y/R159K (**D**). Substrate conformational changes in molecular docking of WT (**E**) and H134Y/R159K (**F**), with oxygen atoms in red and nitrogen atoms in blue.

Tetrasaccharide was docked with the wild-type enzyme and the most active mutant H134Y/R159K. As shown in Figure 6A,B, the substrate was successfully docked into the active cleft, and the substrate molecule binds more tightly to mutant H134Y/R159K. Eight hydrogen bonds were formed between WT and amino acid residues Arg 293, Arg338, Ser292, Arg246, His247, and Asn188 in the substrate molecule (Figure 6C). The residues Arg 293, Arg338, Arg339, His309, Arg246, Asn188, and Ala219 of H134Y/R159K formed eleven hydrogen bonds with the substrate molecule (Figure 6D). Comparing the conformational changes in the substrate molecules in WT and H134Y/R159K, the sulfate group of N-acetyl-D-galactosamine (GalNAc) in the +1 substituent of H134Y/R159K is deflected and shifted from the outer to the active cleft. The inward-facing sulfate group produces three hydrogen bonds with residues Arg339, His309, and Arg246.

## 4. Discussion

Dermatan sulfate is recognized as a potential dietary supplement, and low-molecular-weight dermatan sulfate (LMWDS) is absorbed more easily. Currently, the preparation is experiencing a transition from a chemical route to an enzymatic one due to the numerous benefits including mild conditions, high catalytic efficiency, and selectivity. However, the limited enzymatic activity of chondroitin B lyase is a primary constraint for its current industrial usage. Using protein engineering to enhance the catalytic efficiency of the enzymes has been proven to be a successful approach in modifying the structure of glycosaminoglycan lyases [10,11,12].

In this work, the catalytic efficiency of *Ps*ChonB was improved by modifying the residues around the DS binding pocket using a semirational design strategy. A small mutation library consisting of 13 single-point mutations was constructed using multiple sequence alignment and structural analysis. The enzymatic activities of S90T, N103C, H134Y, and R159K were observed to be considerably higher than the wild type. Among the 13 single-point mutants, Y102F, R104E, R136H, F148Q, P186K, Y198W, and S209H showed a significant decrease compared with that of the wild type. The combinational mutations were conducted on the four single-point mutations, and the *V_max_* of all iterative mutants was found to be increased. One of the mutants, H134Y/R159K, was found to have a *V_max_* of 1392.3 U/mg, which was approximately 3.42 times higher than that of the wild type.

To elucidate the potential mechanism underlying the improved catalytic efficacy of the mutants, molecular docking was employed to demonstrate the interactions between the enzymes and substrate. The residues Arg293, Arg399, Arg246, His309, and Glu220 of the wild-type enzyme formed seven hydrogen bonds with the substrate, with an average bond length of 2.45 Å. The six single-point mutations, such as Y102F, form a reduced number of hydrogen bonds compared with the wild type and lose their interactions with Glu220, as well as Arg293, leading to reduced affinity and decreased activity. S90T, the mutant with increased activity, formed eight hydrogen bonds with the residues in the active pocket with an average length of 2.25 Å. The augmentation in the quantity of hydrogen bonds and the reduced bond length may lead to enhanced catalytic activity. Similarly, the increased numbers of hydrogen bonds generated by mutants N103C, H134Y, and R159K play a role in stabilizing the substrate, hence enhancing the catalytic process and improving the specific activity. The molecular docking analysis of the combinatorial mutation demonstrated that certain combinatorial mutants exhibited an enhanced hydrogen bond contact force with the substrate in comparison with the wild type. The combinatorial mutant H134Y/R159K has nine hydrogen bonds formed with the substrate. In the docking model, the interaction force between Glu308 and Arg225 increased, and a decrease in the average bond length of 0.12 Å was measured compared with the wild type, which may contribute to a substantial increase in its specific activity. Although the four mutants, such as mutant S90T/N103C, did not have any change in the number of hydrogen bonds, their average bond lengths were all shorter than that of WT. The shortening of the hydrogen bond lengths implies that the interactions between the substrate and the surrounding charged amino acid residues are enhanced and the binding is more intense, which is favorable for the extraction of protons on C-5 in the substrate molecule. Simultaneously, the interaction with the hydrophilic amino acid Asn188 may make the intermediate C-5 more stable, thus enhancing the interaction with the substrate. The mutant H134Y/R159K has the lowest affinity of −6.19 kcal/mol, which is 1.03 kcal/mol lower compared with the −5.16 kcal/mol of the wild type (Figure S4). R104E forms only three hydrogen bonds, with the highest binding energy of −2.08 kcal/mol. The mutants S90T, N103C. H134Y, N103C/H134Y, N103C/R159K, and S90T/N103C/H134Y/R159K all form eight hydrogen bonds, with an average affinity of −6 kcal/mol. The increase in the number of hydrogen bonds, as well as the decrease in the affinity, could explain the increase in the viability of the mutants. The specific activity of the mutant H134Y/R159K was much higher than that of *Ps*ChonB (395 U/mg) [35], *Ph*ChonB (84 U/mg) [4,22], and EnCSase (141 U/mg) [23]. This suggests that the mutant has promising potential for industrial and clinical applications.

## 5. Conclusions

In the present work, a mutant H134Y/R159K of *Ps*ChonB was obtained using a semirational design strategy. The mutant exhibited a 321% increase in catalytic activity and a 342% increase in *V_max_* compared with the wild type. Molecular docking and structural analysis indicate that the enhanced activity of the mutant H134Y/R159K may be attributed to an augmentation in the strength of its interaction with the substrate. The mutant possesses evident benefits in the degradation of chondroitin sulfate (CS) or dermatan sulfate (DS), making it more suitable for the production of low-molecular-weight dermatan sulfate (LMWDS) in industry. These results also provide an effective strategy for enzyme remodeling, especially for glycosaminoglycan lyases.

## Figures and Tables

**Table 1:** Kinetic parameters of *Ps*ChonB and mutants around the ligand binding domain.

	*V_max_* (U/mg)	*K*_m_ (mg/mL)
WT	406.72 ± 20.36	0.36 ± 0.03
S90T	895.41 ± 48.66	0.45 ± 0.07
N103C	956.22 ± 74.19	0.38 ± 0.09
H134Y	933.28 ± 40.00	0.31 ± 0.04
R159K	844.08 ± 52.82	0.43 ± 0.08
S90T/N103C	804.01 ± 75.51	0.42 ± 0.12
S90T/H134Y	664.59 ± 70.53	0.57 ± 0.09
S90T/R159K	417.61 ± 20.02	0.20 ± 0.04
N103C/H134Y	586.53 ± 21.46	0.31 ± 0.04
N103C/R159K	800.00 ± 30.20	0.33 ± 0.04
H134Y/R159K	1392.30 ± 103.98	0.37 ± 0. 08
S90T/N103C/H134Y	867.63 ± 61.70	0.29 ± 0.07
S90T/N103C/R159K	837.51 ± 31.82	0.32 ± 0.04
N103C/H134Y/R159K	860.98 ± 33.26	0.39 ± 0.05
S90T/N103C/H134Y/R159K	1051.98 ± 43.09	0.35 ± 0.05

## Data Availability

The original data presented in this work are included in the article and Supplementary Materials, further inquiries can be directed to the corresponding author.

## Supplementary Materials

The following supporting information can be downloaded at: https://www.mdpi.com/article/10.3390/cimb46090591/s1, Table S1: Primer design for *Ps*Chon B mutation; Figure S1: SDS-PAGE images of purified combinatorial mutants; Figure S2: Effect of substrate concentration on the activities of *Ps*Chon B WT and mutants; Figure S3: The internal interactions of the mutants and wild type; Figure S4: The correlation of hydrogen bonds and binding affinity with the substrate.

## References

[1] Linhardt R.J., Galliher P.M., Cooney C.L. (1986). Polysaccharide Lyases. Appl. Biochem. Biotechnol..

[2] Michelacci Y.M., Dietrich C.P. (1976). Structure of Chondroitin Sulfates. Analyses of the Products Formed from Chondroitin Sulfates A and C by the Action of the Chondroitinases C and AC from *Flavobacterium heparinum*. Biochim. Biophys. Acta.

[3] Zhang Z., Su H., Wang X., Tang L., Hu J., Yu W., Han F. (2020). Cloning and Characterization of a Novel Chondroitinase ABC Categorized into a New Subfamily of Polysaccharide Lyase Family 8. Int. J. Biol. Macromol..

[4] Tkalec A.L., Fink D., Blain F., Zhang-Sun G., Laliberte M., Bennett D.C., Gu K., Zimmermann J.J.F., Su H. (2000). Isolation and Expression in *Escherichia coli* of *cslA* and *cslB*, Genes Coding for the Chondroitin Sulfate-Degrading Enzymes Chondroitinase AC and Chondroitinase B, Respectively, from *Flavobacterium heparinum*. Appl. Environ. Microbiol..

[5] Volpi N., Sandri I., Venturelli T. (1995). Activity of Chondroitin ABC Lyase and Hyaluronidase on Free-Radical Degraded Chondroitin Sulfate. Carbohydr. Res..

[6] Sugimura T., Kato F., Mimatsu K., Takenaka O., Iwata H. (1996). Experimental Chemonucleolysis with Chondroitinase ABC in Monkeys. Spine.

[7] Denholm E.M., Lin Y.-Q., Silver P.J. (2001). Anti-Tumor Activities of Chondroitinase AC and Chondroitinase B: Inhibition of Angiogenesis, Proliferation and Invasion. Eur. J. Pharmacol..

[8] Prabhakar V., Capila I., Soundararajan V., Raman R., Sasisekharan R. (2009). Recombinant Expression, Purification, and Biochemical Characterization of Chondroitinase ABC II from *Proteus vulgaris*. J. Biol. Chem..

[9] van der Smissen A., Hintze V., Scharnweber D., Moeller S., Schnabelrauch M., Majok A., Simon J.C., Anderegg U. (2011). Growth Promoting Substrates for Human Dermal Fibroblasts Provided by Artificial Extracellular Matrices Composed of Collagen I and Sulfated Glycosaminoglycans. Biomaterials.

[10] Wang S.-M., Su T.-T., Zhang Q.-D., Guan J.-W., He J., Gu L.-C., Li F.-C. (2019). Comparative Study of Two Chondroitin Sulfate/Dermatan Sulfate 4-O-Sulfatases with High Identity. Front. Microbiol..

[11] Uygun B.E., Stojsih S.E., Matthew H.W.T. (2009). Effects of Immobilized Glycosaminoglycans on the Proliferation and Differentiation of Mesenchymal Stem Cells. Tissue Eng. Part A.

[12] Oberkersch R., Maccari F., Bravo A.I., Volpi N., Gazzaniga S., Calabrese G.C. (2014). Atheroprotective Remodelling of Vascular Dermatan Sulfate Proteoglycans in Response to Hypercholesterolaemia in a Rat Model. Int. J. Exp. Pathol..

[13] Godoy J.A.P., Carneiro G.D., Sielski M.S., Barbosa G.O., Werneck C.C., Vicente C.P. (2015). Combined Dermatan Sulfate and Endothelial Progenitor Cell Treatment: Action on the Initial Inflammatory Response after Arterial Injury in C57BL/6 Mice. Cytotherapy.

[14] Bucay V., Gold M.H., Andriessen A. (2020). Low Molecular Weight Heparan Sulfate Containing Facial Skin Care for Reducing Inflammation and Restoring Aged-Skin Homeostasis. J. Cosmet. Dermatol..

[15] Ma C., Yu M., Huang Z., Wang J., Zhao X., Kang C., Xu H., Wang Y., Hou H. (2021). Oral Administration of Hydrolysates of Cartilage Extract in the Prevention of Osteoarthritis. J. Funct. Foods.

[16] Chopra A.S., Lordan R., Horbańczuk O.K., Atanasov A.G., Chopra I., Horbańczuk J.O., Jóźwik A., Huang L., Pirgozliev V., Banach M. (2022). The Current Use and Evolving Landscape of Nutraceuticals. Pharmacol. Res..

[17] Wang H., Betti M. (2018). Supplementation of Chondroitin Sulfate-Oligosaccharides in Skim Bovine Milk Improves Fe Uptake in a Human Intestinal Caco-2 Cell Line. J. Funct. Foods.

[18] Wu J., Ji Y., Su N., Li Y., Liu X., Mei X., Zhou Q., Zhang C., Xing X. (2016). Establishment of Chondroitin B Lyase-Based Analytical Methods for Sensitive and Quantitative Detection of Dermatan Sulfate in Heparin. Carbohydr. Polym..

[19] Yu X., Liu J., Wan J., Zhao L., Liu Y., Wei Y., Ouyang Z. (2020). Cloning, Prokaryotic Expression, and Enzyme Activity of a UDP-Glucose Flavonoid 3-o-Glycosyltransferase from Mulberry (*Morus alba* L.) Leaves. Pharmacogn. Mag..

[20] Tian X., Chen Y., Peng Z., Lin Q., Sun A. (2023). NEDD4 E3 Ubiquitin Ligases: Promising Biomarkers and Therapeutic Targets for Cancer. Biochem. Pharmacol..

[21] Ullah H., Di Minno A., Piccinocchi R., Buccato D.G., De Lellis L.F., Baldi A., El-Seedi H.R., Khalifa S.A.M., Piccinocchi G., Xiao X. (2023). Efficacy of Digestive Enzyme Supplementation in Functional Dyspepsia: A Monocentric, Randomized, Double-Blind, Placebo-Controlled, Clinical Trial. Biomed. Pharmacother..

[22] Yamagata T., Saito H., Habuchi O., Suzuki S. (1968). Purification and Properties of Bacterial Chondroitinases and Chondrosulfatases. J. Biol. Chem..

[23] Zhang Q., Lu D., Wang S., Wei L., Wang W., Li F. (2020). Identification and Biochemical Characterization of a Novel Chondroitin Sulfate/Dermantan Sulfate Lyase from *Photobacterium* sp.. Int. J. Biol. Macromol..

[24] Zhao J.C., Mu Y.L., Gu X.Y., Xu X.N., Guo T.T., Kong J. (2022). Site-Directed Mutation of β-Galactosidase from Streptococcus Thermophilus for Galactooligosaccharide-Enriched Yogurt Making. J. Dairy Sci..

[25] Lyu J., Zhang J., Zhu J., Chen S., Han T., Zhang Y., Gao R., Xie G., Guo Z. (2022). Molecular Dynamics Simulation Guided Distal Mutation of Thermotoga Naphthophila β-Glucosidase for Significantly Enhanced Synthesis of Galactooligosaccharides and Expanded Product Scope. Int. J. Biol. Macromol..

[26] Ratananikom K., Choengpanya K., Tongtubtim N., Charoenrat T., Withers S.G., Kongsaeree P.T. (2013). Mutational Analysis in the Glycone Binding Pocket of Dalbergia Cochinchinensis β-Glucosidase to Increase Catalytic Efficiency toward Mannosides. Carbohydr. Res..

[27] Zhu Y., Peng J., Zhao Y., Wu M., Chen S., Shao J., Wang X., Xia G., Shen Y. (2023). Obtaining Acid-Sensitive Prosaikogenin F by Enzymatic Hydrolysis of Saikosaponin A. Pharmacogn. Mag..

[28] Su W.-B., Zhu C.-Y., Zhou H.-P., Gao J., Zhang Y.-W. (2022). A Single Site Mutation Significantly Improves the Thermostability and Activity of Heparinase I from *Bacteroides eggerthii*. Biocatal. Biotransform..

[29] Zhou H.-P., Wang D.-R., Xu C.-L., Zhang Y.-W. (2023). Combination of Engineering the Substrate and Ca2+ Binding Domains of Heparinase I to Improve the Catalytic Activity. Prep. Biochem. Biotechnol..

[30] Huang J.-Y., Fan X.-M., Yu S., Zhang J.-Y., Gao J., Zhang Y.-W. (2024). Engineering the Terminal Regions of Chondroitinase AC to Improve the Thermostability and Activity. Mol. Catal..

[31] Sun H., Cheng Y., Zhao L., Cao R. (2024). Improvement of the Catalytic Performance of Chitosanase Csn-PD from Paenibacillus Dendritiformis by Semi-Rational Design. Int. J. Biol. Macromol..

[32] Luo J., Gu S., Zhu J., Ro K.-S., Zhao L., Du L., Xie J., Wei D. (2024). Screening, Cloning and Expression of β-Galactosidase from Lactic Acid Bacteria and Semi-Rational Design for the Improvement of Transgalactosylation Activity. Process Biochem..

[33] Jiang S., Zhang Z., Gu Q., Yu X. (2024). Semi-Rational Design for Enhancing Thermostability of Culex Pipiens Acetylcholinesterase and Sensitivity Analysis of Acephate. Sci. Total Environ..

[34] Li M., Zhang T., Li C., Gao W., Liu Z., Miao M. (2024). Semi-Rationally Designed Site-Saturation Mutation of Helicobacter Pylori α-1,2-Fucosyltransferase for Improved Catalytic Activity and Thermostability. Int. J. Biol. Macromol..

[35] Xu Y.-Y., Tian M., Tang Y.-L., Han K.-K., Yu S., Ma X.-L., Zhang Y.-W. (2023). A Highly Active and Stable Chondroitin B Lyase from Pedobacter Schmidteae: Cloning, Expression, and Characterization. Biocatal. Agric. Biotechnol..

[36] Bradford M.M. (1976). A Rapid and Sensitive Method for the Quantitation of Microgram Quantities of Protein Utilizing the Principle of Protein-Dye Binding. Anal. Biochem..

