# Supplementary material for: Engineering of Substrate-Binding Domain to Improve Catalytic Activity of Chondroitin B Lyase with Semi-Rational Design
Source: Curr Issues Mol Biol. 2024 Sep 6;46(9):9916–27. doi: 10.3390/cimb46090591 (PMC11429652; doi:10.3390/cimb46090591)
Supplement: Supplementary file 1 [file cimb-46-00591-s001.zip › cimb-3155180-supplementary.pdf]

Supplementary Materials:

Table S1. Primer design for *Ps*Chon B mutation.

Primers	Sequences **(5'to 3' direction)**	Tm(°C)
S90T-F	GCTATTCAGGCATGGAAAACCCAGGGCCGGGTCTGGTTG	74.2
S90T-R	CAACCAGACCCGGCCCGTGGGTTTTCCATGCCTGAATAGC	74.2
F148Q-F	TCACCGATAAAATCACTCAGGATCAGGTAATCCTGAC	63.6
F148Q-R	GTTCAGGTTGATTACCTGATCCTGAGTTTTATCGGTGA	63.6
R159K-F	CCTGAACAACACCGCGAAAGCGATCAAAGACGGTAG	68.0
R159K-R	CTACCGTCTTTGATCGCTTTCGCGGTGTTGTTCAGG 3'	68.0
P186K-F	CAGCAACCCGCAGAAAAAAGGTAACGCGGGCGGTG	72.6
P186K-R	CACCGCCCGCGTTACCTTTTTTCTGCGGGTTGCTG	72.6
Y198W-F	GTATCCGCATTGGTTACTGGCGCAACGACATCGGCCG	67.2
Y198W-R	CGGCCGATGTCGTTGCGCCAGTAACCAATGCGGATAC	67.2
Y102H-F	GTTGCTATCTACGGTTCCCACAACCGTATCACCGCTTG	62.3
Y102H-R	CAAGCGGTGATACGGTTGTGGGAACCGTAGTAGCAA	62.3
N103C-F	CTATCTACGGTTCCTACTGCCGTATCACCGCTTGC	67.4
N103C-R	GCAAGCGGTGATACGGCAGTAGGAACCGTAGATAG	67.4
R104E-F	CTACGGTTCCTACAACGAGATCACCGCTTGCGTG	60.5
R104E-R	CACGCAAGCGGTGATCTCGTTGTAGGAACCGTAG	60.5
H134Y-F	GATGGCAAAGTTCCGCAGTACTGCCGTATTGATCACT	67.2
H134Y-R	CAGTGATCAATACGGATGTACTGCGGAACTTTGCCAT	67.2
R136H-F	CAAAGTTCCGCAGCATTGCCACATTGATCACTGCTCTT	68.2
R136H-R	GTGAAAGAGCAGTGATCAATGTGGCAATGCTGCGGC	68.2
S209H-F	CCGCTGCCTGGTTGACCACAACTTATTTATGCG	66.3
S209H-R	CGCATAAATAAGTTGTGGTCAACCAGGCAGCGG	66.3
Y232F-F	CTCAAGAAAACGTGTACTTCGGCAACACTTACCTGAA	62.5
Y232F-R	GTTCAGGTAAGTGTTGCCGAAGTACACGTTTTCTTGA	62.5
G437D-F	CAAAGGTATCACCGACAAACCGCTGTCCTGG	66.5
G437D-R	CCAGGACAGCGGTCTGTCGGTGATACCTTTG	66.5

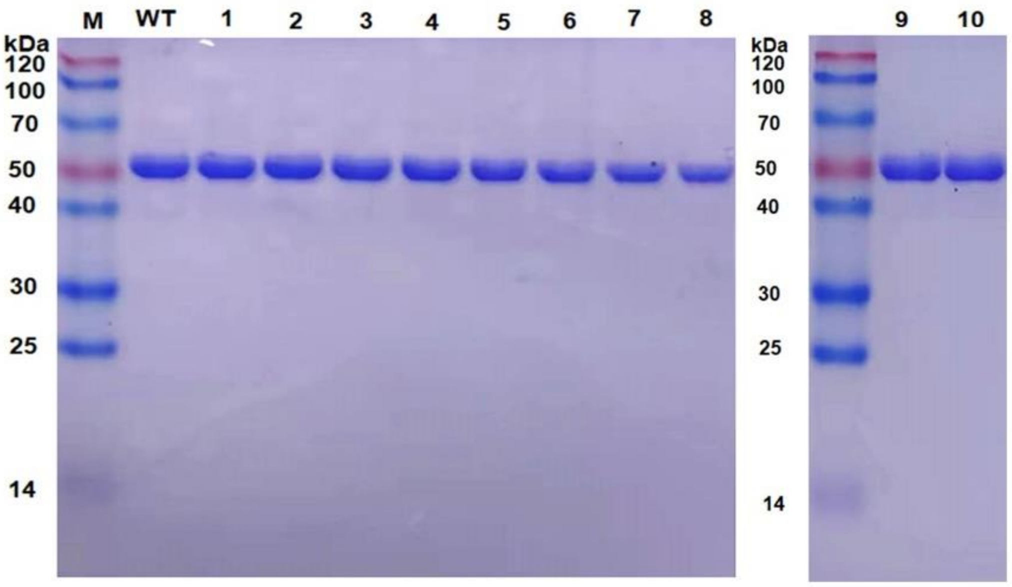

Figure S1. SDS-PAGE images of purified combinatorial mutants. (Lane M, protein marker; Lane 1, S90T/N103C; Lane 2, S90T/H134Y; Lane 3.S90T/R159K; Lane 4, N103C/H134Y; Lane 5, N103C/R159K; Lane 6, H134Y/R159K; Lane 7, S90T/N103C/H134Y; Lane 8, S90T/N103C/R159K; Lane 9, N103C/H134Y/R159; Lane 10, S90T/N103C/H14Y/R159K).

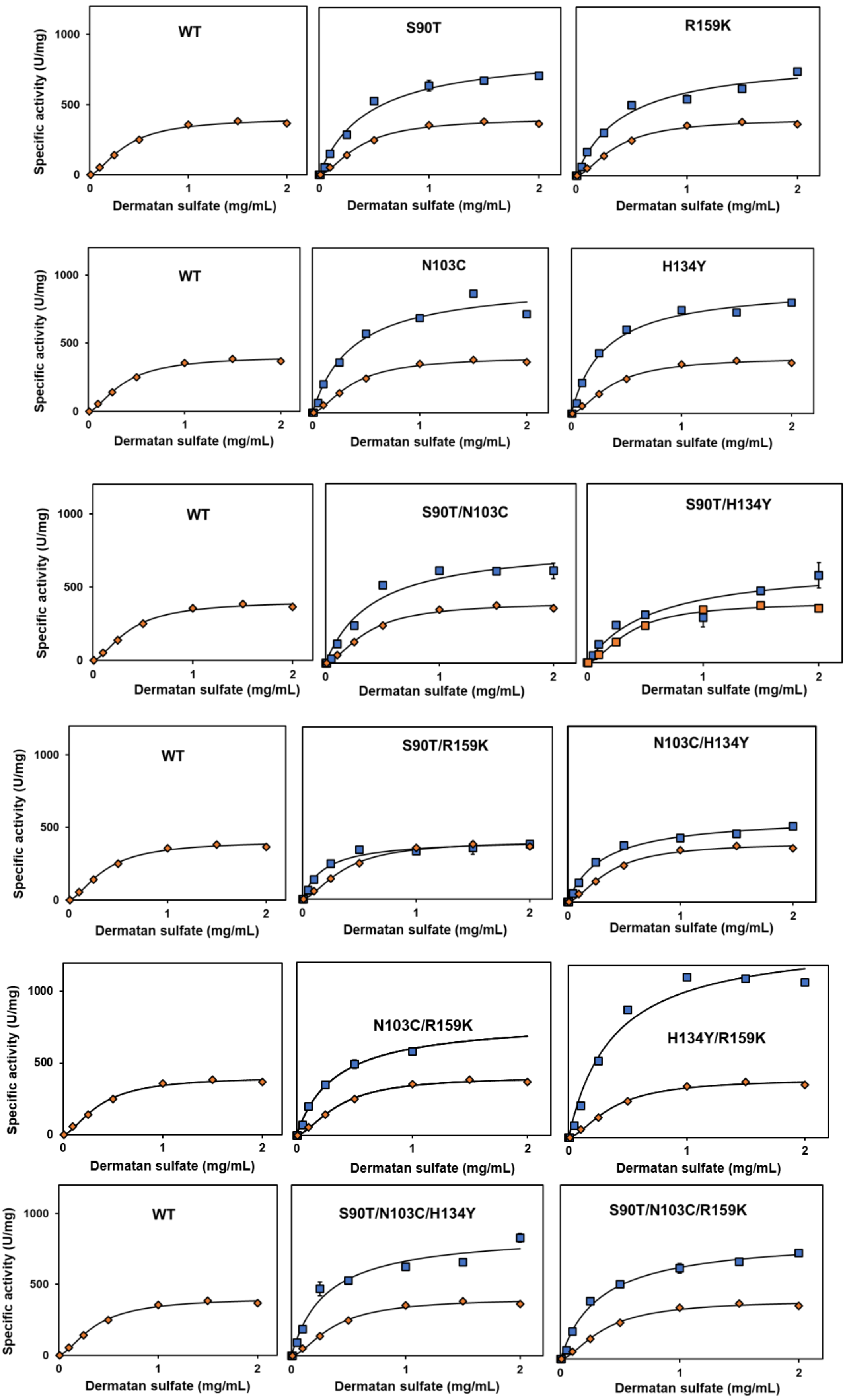
WT
S90T
R159K
N103C
H134Y
S90T/N103C
S90T/H134Y
S90T/R159K
N103C/H134Y
N103C/R159K
H134Y/R159K
S90T/N103C/H134Y
S90T/N103C/R159K
Specific activity (U/mg)
Dermatan sulfate (mg/mL)
0
500
1000
1
2

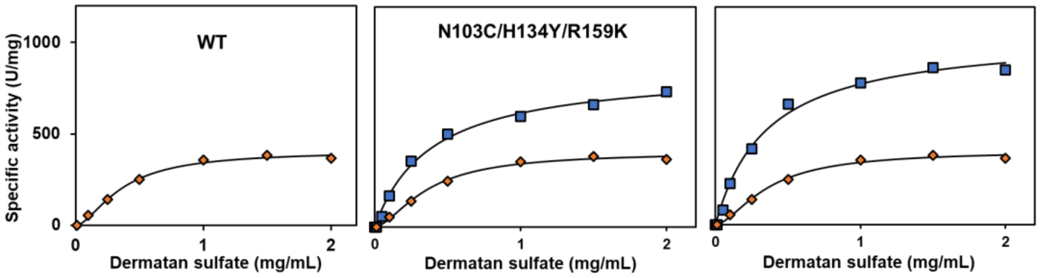

Figure S2. Effect of substrate concentration on the activities of *Ps*Chon B WT and mutants

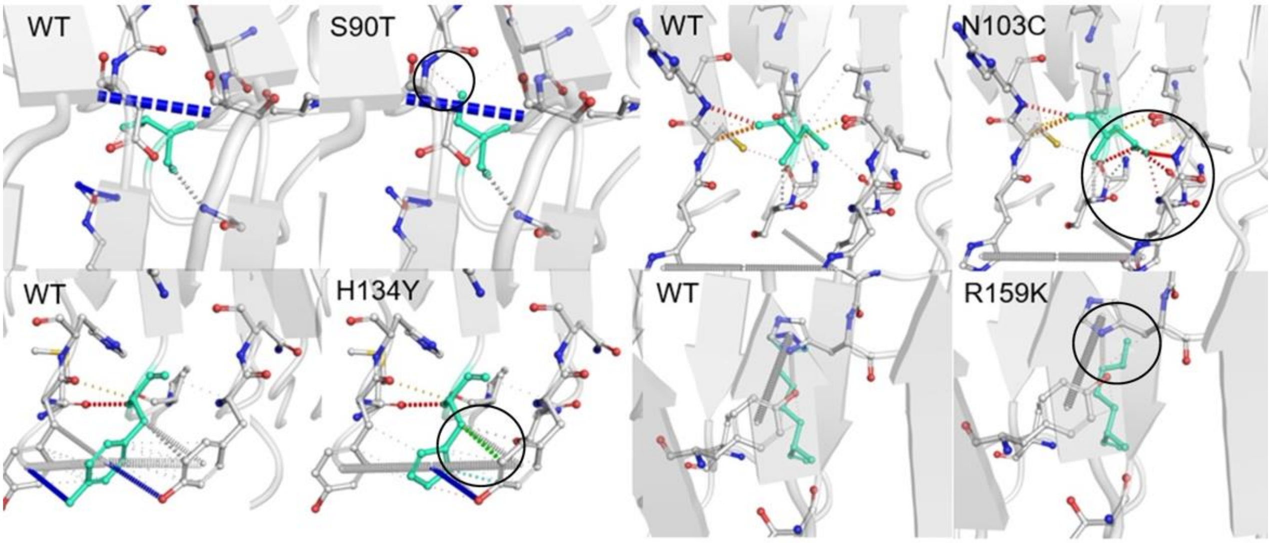

Figure S3. The internal interactions of the mutants and wild type. Different interactions were colored as follows: hydrogen bonds (red), weak hydrogen bonds (orange), ionic bonds (yellow), hydrophobic forces between neighboring molecules (gray), hydrophobic bonds under de Waal forces (green), and the interaction between two amide bonds (blue).

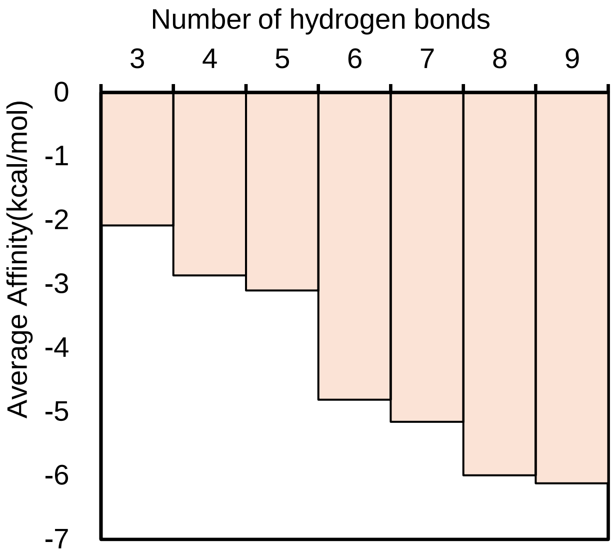

Figure S4. The correlation of hydrogen bonds and binding affinity with the substrate.
